# Development and Validation of an LC-MS/MS Method for the Quantitation of JNJ-64619178 (JNJ) in Mouse Plasma: Characterization of In Vitro and In Vivo Pharmacokinetic Properties

**DOI:** 10.3390/molecules31091396

**Published:** 2026-04-23

**Authors:** Nusrat Ahmed, Pratiksha Kshirsagar, Ling Ding, Daryl J. Murry, Nagendra K. Chaturvedi, Yashpal S. Chhonker

**Affiliations:** 1 Clinical Pharmacology Laboratory, Department of Pharmacy Practice and Science, University of Nebraska Medical Center, Omaha, NE 68198, USA; nusrat.9208@gmail.com (N.A.); pkshirsagar@unmc.edu (P.K.); ling.ding@unmc.edu (L.D.); dj.murry@unmc.edu (D.J.M.); 2 Division of Hematology/Oncology, Department of Pediatrics, University of Nebraska Medical Center, Omaha, NE 68198, USA

**Keywords:** medulloblastoma, LC-MS/MS bioanalysis, validation, pharmacokinetics

## Abstract

Overexpression of protein arginine methyltransferase 5 (PRMT5) is pivotal in MYC-driven primary medulloblastoma tumors, suggesting PRMT5 as a potential therapeutic target. JNJ, a potent PRMT5 inhibitor currently in clinical trials, notably for non-Hodgkin lymphoma and lung cancer, was evaluated in this study. We report a validated LC–MS/MS bioanalytical method for quantifying JNJ in plasma and tissue matrices. The method demonstrated acceptable sensitivity, selectivity, and robustness in accordance with regulatory guidelines. The assay was linear over the range 0.2–500 ng mL^−1^ (r^2^ = 0.99), with plasma recovery exceeding 84% using only 100 µL of sample. Precision (%RSD < 15%) and accuracy (~91–108%) were within acceptable limits. JNJ showed >94% plasma protein binding and moderate Caco-2 permeability (3.4 ± 0.4 × 10^−6^ cm s^−1^). Hepatic intrinsic clearance was higher in mouse liver microsomes than in human (41 ± 19 vs. 7 ± 0.6 mL min^−1^ kg^−1^). Following oral dosing in mice (10 mg kg^−1^), T_max_ was 30 min with a C_max_ of 2781 ± 1033 ng mL^−1^. Oral bioavailability was low (15%). The validated method was successfully applied to in vitro and in vivo studies and will guide dosing in animal models of medulloblastoma.

## 1. Introduction

Medulloblastoma (MB) is the most common malignant pediatric brain tumor and accounts for around 12 to 25% of all central nervous system tumors in children [1,2,3]. Current treatment strategies for MB include maximal safe surgical resection, chemotherapy, and craniospinal irradiation, resulting in an increase in the survival rate among patients [4]. Nevertheless, survivors often suffer from long-term side effects of therapy, which impact their quality of life. Among four distinct molecular subgroups of MB, namely, wingless (WNT), Sonic Hedgehog (SHH), Group 3, and Group 4, Group 3 tumors typically predominate in the pediatric age spectrum and represent approximately 45% of MB tumors in the infant population [5,6]. Group 3 MB tumors are driven by inferior outcomes with an overall survival rate of <60% [5]. In a subset of Group 3 tumors, tumorigenesis based on gene expression reveals that amplification or overexpression of the MYC oncogene is a hallmark of Group 3 tumors [7]. Seventy percent of patients with aberrant MYC expression frequently experience treatment resistance, are associated with early metastasis, and carry the worst prognosis [8]. Moreover, MYC-driven MB is metastatic, and patients suffer from poor clinical outcomes even after multimodal treatments [9,10]. Thus, identifying new targeted therapeutics in the MB treatment paradigms is of critical need.

In mammals, the epigenetic enzyme protein arginine methyltransferase 5 (PRMT5) is a primary type II arginine methyltransferase that plays a key role in malignant cell proliferation and differentiation by regulating the methylation of cellular proteins [11]. In MYC-driven MB, PRMT5 stabilizes the MYC protein and enhances the cells growth. Our group observed poor survival of Group 3 MB patients with elevated PRMT5 expression, and studies with PRMT5 knockdown revealed potential suppression of MYC expression [12]. Phase I studies of JNJ revealed a dose-dependent plasma profile alongside effective target inhibition in advanced solid tumors [13,14]. However, a detailed description of the analytical method, validation parameters, or full methodological references has not been provided. In addition, the ability of JNJ to cross the blood–brain barrier and its potential role in the therapy of MB in terms of safety and efficacy have not been described. There is limited information in the literature regarding the in vitro properties of JNJ (i.e., plasma protein binding and metabolic stability), and there is only one published study characterizing the in vivo pharmacokinetic (PK) behavior of JNJ in mice [15]. However, there is no description of the analytical method used for quantification, nor are details published elsewhere.

Based on these observations, our current study addresses these gaps and aims to assess the in vitro and in vivo PK characterization of JNJ along with the ability of JNJ to cross the blood–brain barrier (BBB), a critical determinant for drug therapy for medulloblastoma. Liquid chromatography–mass spectrometry (LC-MS/MS) is the method of choice for bioanalytical quantitation in terms of high selectivity, specificity, sensitivity, and accuracy [16,17]. Thus, here we sought to develop and validate a reliable LC-MS/MS method for the determination of JNJ in biological matrices, ensuring high analytical precision and reliability. Furthermore, we implemented in vitro drug metabolism and PK (DMPK) screening to assess the physicochemical, metabolic and distribution properties of JNJ, alongside investigating in vivo PK and CNS penetration studies at the preclinical stage. Thus, the present work extends beyond earlier studies by providing a detailed, robust LC-MS/MS validated assay that is easily reproducible. While the previous study [15] reported PK properties (clearance and bioavailability) in mice, our integrated approach allows more complete PK characterization and deeper interpretation of absorption, distribution, and elimination processes. Preclinical studies are also critical to optimize the dosing regimen and routes of administration while providing valuable preliminary data on efficacy, thus enhancing the likelihood of success in clinical trials. These findings would leverage understanding of JNJ in the biological system and would support the continued clinical evaluation of JNJ as a PRMT5 inhibitor in cancer treatment.

## 2. Results

### 2.1. Method Development

In our study, we developed and optimized an LC-MS/MS method for quantifying JNJ in biological matrices. First, for the MS condition, the electrospray ionization source (ESI) positive mode was chosen over the atmospheric pressure chemical ionization (APCI) mode based on the signal intensity of the precursor ion for both the analyte and the internal standard (IS). The precursor ion peaks for JNJ and IS were observed at *m*/*z* values of 484 and 250, respectively, corresponding to the protonated molecule, [M+H]+. A multiple-reaction-monitoring (MRM) auto-optimization step was carried out to obtain the most abundant precursor-to-product ions with the highest sensitivity. MRM transitions of JNJ and IS are represented as an MRM pair (quantifier and qualifier) in Table 1.

The chromatographic condition was finalized based on good retention and peak shapes of both the analyte and IS on the selected Synergy polar-RP80 Å (150 × 2 mm, 4 µm, Phenomenex, Torrance, CA, USA) column. The retention times of JNJ and oxibendazole were observed at 3.7 and 5 min, respectively, and were well separated.

### 2.2. Method Validation

**Figure 1:**
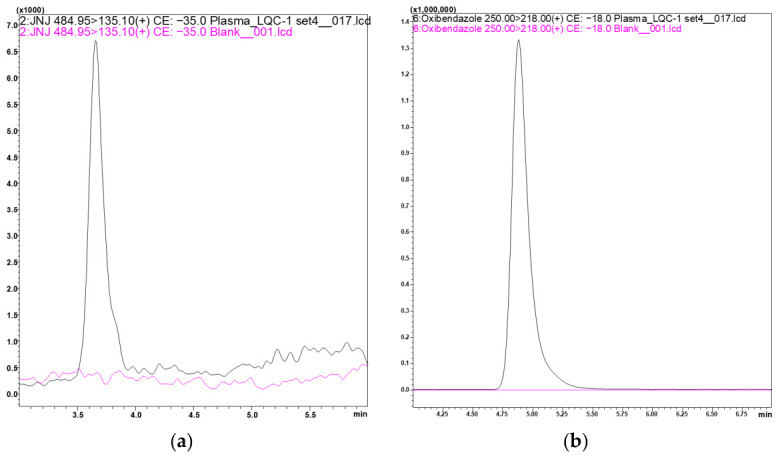
Representative overlay chromatograms: (**a**) blank plasma and JNJ spiked in plasma at LQC (retention time 3.7 min); (**b**) blank plasma and IS spiked in plasma (retention time 5 min).

The developed method represented good sensitivity, selectivity, and robustness aligned with the regulatory guideline’s requirements. Endogenous interfering peaks were not observed at the retention time of the analyte and IS, represented in the overlay chromatogram with blank plasma and JNJ spiked at a low quality control (LQC) concentration (Figure 1).

The calibration curve was best fitted using a linear equation of Y = 0.023X + 0.006 using a 1/X^2^ weighing factor with an r^2^ value of 0.99.

The mean % extraction recoveries of JNJ at three quality control (QC) levels—LQC, medium quality control (MQC), and high quality control (HQC)–in mouse plasma were 84 ± 8, 112 ± 3, and 99 ± 9, respectively. With the developed sample preparation method, IS recoveries were also consistent with a value of 98 ± 14, 115 ± 13, and 113 ± 6, respectively, at LQC, MQC, and HQC. The calculated matrix effect for JNJ and IS at the specified level was found to be ≤15% in mouse plasma.

The intra-day and inter-day accuracy determined by % bias and precision determined by percent relative standard deviation (% RSD) were within the FDA-specified acceptable range for the lower limit of quantitation (LLOQ), LQC, MQC, and HQC and are represented in Table 2.

JNJ is depicted as stable in all bioanalytical storage conditions mentioned in the method section, and results are represented in Table 3.

### 2.3. Stability in Plasma and Brain Matrix

To investigate the enzymatic degradation of JNJ in plasma or other matrices (brain), we performed a stability study of JNJ in mouse plasma and brain homogenate. The JNJ concentration remaining (%) in mouse plasma and brain matrices for approximately 5 h was almost 100% in triplicate. This result indicated that JNJ did not show significant degradation in the plasma or brain, suggesting relative stability under the tested experimental conditions [18]. This important parameter guided our further bioanalytical assay design.

### 2.4. In Vitro PK Studies

#### 2.4.1. Microsomal Stability

**Figure 2:**
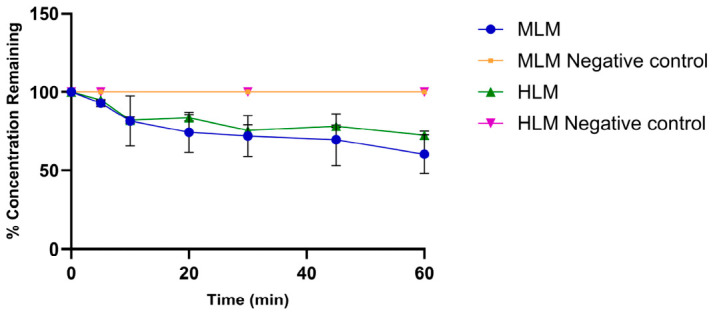
Metabolic stability of JNJ in mouse (MLMs) and human liver microsomes (HLMs); (n = 3).

Microsomal metabolic stability determines cytochrome P450 (CYP)-mediated metabolism of the molecules in the presence of nicotinamide adenine dinucleotide phosphate (NADPH)-fortified liver microsomes from humans and other species [18]. We have observed that JNJ has low metabolic clearance in mouse (MLMs) and human liver microsomes (HLMs). The percent remaining concentration of JNJ after 1 h incubation was 60 ± 12 and 72 ± 2%, respectively, in MLMs and HLMs (Figure 2). The metabolic stability of JNJ in mouse and human liver microsomes expresses that it does not have any phase-I-mediated major metabolites that could be active or cause toxicity. The calculated mean microsomal intrinsic clearance (CL_int_) values in MLMs and HLMs were 0.02 ± 0.01 and 0.01 ± 0.001 mL min^−1^ mg^−1^ protein, respectively. The mean hepatic intrinsic clearance (CL_H,int_) in MLMs and HLMs were 41 ± 19 and 7 ± 0.5 mL min^−1^ kg^−1^ body weight, respectively, as reported in Table 4, which signifies that the rate of disappearance was comparatively lower in HLMs compared to MLMs. The mean predicted in vivo hepatic clearance (CL_H,predict_) was calculated using in vitro scaling and was 1.6 ± 0.7 mL min^−1^ kg^−1^ body weight in mice.

#### 2.4.2. Plasma and Brain Protein Binding

Determining the free drug (unbound) concentration in plasma is an important point to consider, as only the unbound drug is available to exert its pharmacological action and undergo subsequent elimination [19]. JNJ exhibited very high protein binding to mouse plasma and brain tissue, with a value of >94% represented in Table 5. The free fraction parameter of JNJ in the plasma and brain obtained from the protein binding study was utilized further to estimate plasma and brain disposition in in vivo PK studies, which is a more reliable approach.

#### 2.4.3. Kinetic Solubility Study

To assess JNJ’s aqueous solubility, we performed a kinetic solubility study and found that JNJ has low aqueous solubility with a value of 3 ± 0.6 µg mL^−1^. The low aqueous solubility predicted that it would limit the absorption of JNJ from the gastrointestinal tract, challenging its oral bioavailability.

#### 2.4.4. Blood-to-Plasma Ratio (R_b/p_) Study

In the blood plasma portioning study, we observed that the R_b/p_ of JNJ was approximately 0.70, and R_b/p_ < 1 suggests that JNJ did not partition into red blood cells; this plasma data will be useful for further PK analysis.

#### 2.4.5. Parallel Artificial Membrane Permeability Assay (PAMPA) Study

To evaluate JNJ’s in vitro permeability properties, we conducted an artificial-membrane-based permeability assay. This assay provides critical information on effective routes of administration (i.e., oral or intravenous). In the PAMPA experiment, JNJ showed moderate apparent permeability (P_app_) with a value of 7 ± 4 (×10^−6^) cm s^−1^.

#### 2.4.6. Caco-2 Permeability Study

To predict the intestinal absorption pattern of JNJ in a reliable manner, we also performed a cell-monolayer-based permeability assay using a Caco-2 cell monolayer. In the Caco-2 cell permeability assay, JNJ exhibited moderate P_app_ values of 3 ± 0.4 (×10^−6^) cm s^−1^ in the absorptive direction (apical to basal). The P_app_ result obtained from Caco-2 is in good agreement with PAMPA, suggesting JNJ is not a substrate of active efflux transporter (e.g., P-gp or BCRP).

### 2.5. In Vivo PK and Biodistribution Studies

**Figure 3:**
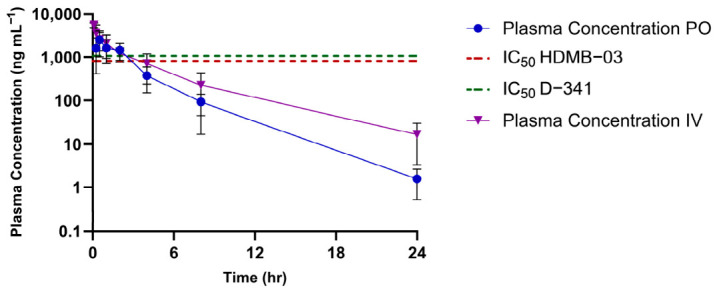
Plasma concentration vs. time profile following a single PO (10 mg kg^−1^) and IV (2.5 mg kg^−1^) dose of JNJ in balb/c mice (mean ± SD, n = 4 for PO and 6 for IV at each time point).

We performed in vivo PK analysis for JNJ by 2.5 mg kg^−1^ IV and 10 mg kg^−1^ PO routes of administration in healthy male balb/c mice. We could quantify a dynamic range of concentrations in in vivo PK samples with our established LC-MS/MS method. Non-compartmental analysis (NCA) was performed using Phoenix software version 8.3 to estimate PK parameters from the observed plasma concentration–time profile data. The plasma PK profile of JNJ following a single IV and PO dose is shown in Figure 3. Following the oral administration of JNJ (10 mg kg^−1^), it showed a rapid onset of T_max_ of ~30 min, and the maximum plasma concentration (C_max_) achieved was 2781 ± 1033 ng mL^−1^, which was ~3 times higher than the IC_50_ of JNJ in the HD-MB cell line (1.7 µM or 821 ng mL^−1^). The NCA PK parameter data are represented in Table 6.

**Figure 4:**
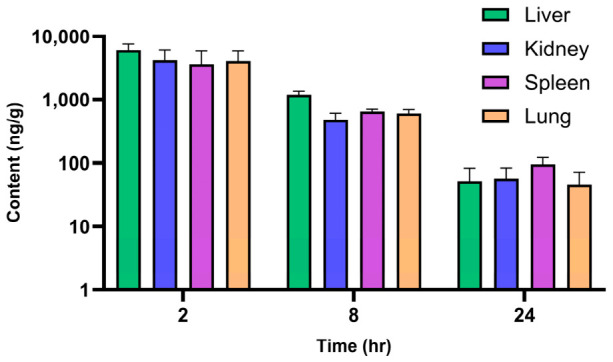
JNJ tissue distribution profile following a single PO (10 mg kg^−1^) dose of JNJ in balb/c mice (mean ± SD, n = 4 at each time point).

Following IV dosing, plasma clearance (CL) was 0.23 L h^−1^ kg^−1^, and the volume of distribution (V_z_) was 1.2 L kg^−1^. The in vivo clearance was observed as a value of 0.23 L h^−1^ kg^−1^, which is approximately only 6% of the mice hepatic blood flow (3.6 L h^−1^ kg^−1^) [20]. This result depicted that JNJ has slow clearance, which was consistent with the in vitro microsomal data [20]. The volume of distribution value was ~1.6 times the total body water in the mouse tissue (68.6% of body weight) [21]. This indicates a substantial distribution of JNJ into tissues. After a single PO dose, the mean terminal half-life was 2.6 h, and the oral bioavailability achieved was low (15%). Following PO administration, the biodistribution result confirmed JNJ’s substantial tissue distribution pattern (Figure 4). Although JNJ contents in all evaluated tissues (liver, kidney, lung and spleen) exceeded the IC_50_, it was rapidly cleared from these tissues, with minimal levels remaining at 24 h (Table 7).

**Figure 5:**
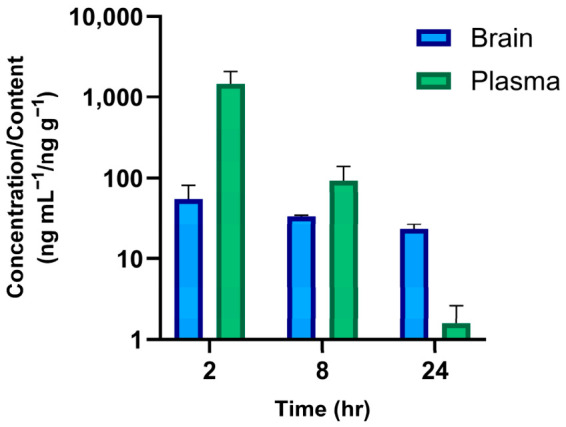
JNJ brain distribution profile following a single PO (10 mg kg^−1^) dose of JNJ in balb/c mice (mean ± SD, n = 4 at each time point).

The unbound brain-to-plasma partition coefficient (K_p,uu,brain_) is a key parameter related to brain exposure based on unbound concentration in the brain [22]. Although K_p,uu,brain_ at 2 h was 0.08, the compound was well-retained in the brain environment even after 24 h with a value of 33.3 (Figure 5 and Table 8). The unbound brain content achieved after JNJ administration was below the IC_50_ value, suggesting the need for a suitable drug delivery system to improve JNJ brain penetration for the treatment of MB.

## 3. Discussion

**Figure 6:**
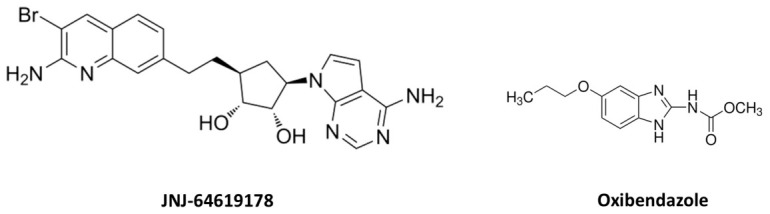
The chemical structure of JNJ-64619178 and oxibendazole (IS).

This study developed and validated a sensitive and reliable bioanalytical LC-MS/MS method for the quantification of JNJ in plasma and tissue matrices. Our goal was to provide a robust bioanalytical method that can be further used in the interpretation of in vitro and in vivo PK findings. To the best of our knowledge, this is the first reported bioanalytical LC-MS/MS method for quantifying JNJ in biological matrices, presented as a comprehensive analytical framework, and we hope it will serve as a valuable resource in its continued clinical evaluation. In our developed method, we selected the ESI positive ion mode, which resulted in higher signal intensity and improved sensitivity for JNJ and IS compared to the APCI mode. Oxibendazole was selected as the IS due to its comparable basic properties and ionization efficiency in positive ESI mode, as well as similar chromatographic retention behavior, which ensured consistent extraction recovery and minimal matrix interference. The analytes JNJ and oxibendazole both possess basic and polar functional groups (Figure 6), and the positive ESI mode facilitated the formation of stable protonated molecule peaks [23,24]. Product ions generated through the controlled collision-induced dissociation of the protonated molecule in the MRM auto-optimization process ensured a highly specific MRM identifier pair (Table 1). Obtaining the most abundant precursor-to-product ions in MRM transitions of JNJ improves analytical selectivity and sensitivity by reducing interference from endogenous matrix components [25].

Chromatographic separation was carried out using the selected Synergy polar-RP80 Å (150 × 2 mm, 4 µm, Phenomenex, Torrance, CA, USA) column, leveraging the specific properties of a polar RP column. This stationary phase is most used for its enhanced polar interactions and reversed-phase retention mechanisms, leading to better retention and peak symmetry (Figure 1). The observed retention times of approximately 3.7 min for JNJ and 5 min for oxibendazole indicate effective chromatographic separation. The reproducibility of retention times for both JNJ and oxibendazole across analytical runs further ensured method reliability and the minimization of co-elution risk. Together, the developed chromatographic and mass spectrometric conditions contributed to the robustness of the bioanalytical method.

The validated LC-MS/MS method had an LLOQ of 0.2 ng mL^−1^ using only 100 µL of sample, indicating high analytical sensitivity. Such sensitivity was crucial for accurately measuring the low drug concentration at later sampling points in the subsequent in vivo PK studies. The calibration curve was linear in a range of 0.2–500 ng mL^−1^, with a determination coefficient of 0.99, which is in line with the regulatory guidance and further supports accurate quantification across a broad concentration range. The low intercept value in the linear equation of Y = 0.023X + 0.006 suggested limited bias at the lowest concentrations. We developed a solid-phase extraction (SPE) method that resulted in consistent % extraction recoveries (>84%) across QC levels for both analyte and IS. Thus, the sample preparation method was efficient and suitable for routine analysis. Recovery values exceeding 100% at MQC for both the analyte and IS indicate matrix-induced ion enhancement. But this observation is consistent with the matrix effect evaluation (~12% at MQC). The calculated matrix effect was low (≤15%) for JNJ and IS at the specified level, indicating that ion suppression or enhancement was well controlled and did not adversely affect assay performance. Both intra-day and inter-day accuracy (% bias) and precision (% RSD) were within the FDA-specified acceptable limits, confirming that the method is reproducible over time and across analytical runs [26]. This reproducibility is particularly relevant for preclinical PK studies, where samples are often analyzed over extended study durations and across multiple analytical batches. It can reduce analytical variability as a confounding factor in PK parameter estimation. The depicted stability of JNJ under all tested bioanalytical storage conditions further validated the suitability of the assay for preclinical studies. This is especially important for the accurate quantification of compounds evaluated in tissue distribution and brain exposure studies, where sample processing and storage may be lengthy and complex.

In vitro PK studies showed that JNJ has limited CYP-mediated phase I metabolism, similar to other structurally related PRMT5 inhibitors with slow intrinsic clearance in liver microsomes [27]. However, the predicted hepatic clearance (1.6 mL min^−1^ kg^−1^) underestimated the observed plasma clearance (3.8 mL min^−1^ kg^−1^ equivalent to 0.23 L h^−1^ kg^−1^) by approximately 2.3-fold. This level of difference is commonly seen with IVIVE predictions [28] and suggests that, although microsomal data capture intrinsic metabolic trends, additional in vivo processes—such as extrahepatic metabolism or transporter-mediated processes—may also contribute to the higher observed clearance. We found that JNJ exhibited high plasma and brain protein binding (>94%), as summarized in Table 5. Consistent with its physicochemical profile, JNJ has low aqueous solubility and moderate permeability in both PAMPA and Caco-2 assays. The agreement between PAMPA and Caco-2 data further suggests that active efflux transporters do not play a major role in limiting the intestinal absorption of JNJ. Despite this, the low oral bioavailability (~15%) observed in vivo is unlikely to be primarily driven by permeability limitations. Instead, solubility-limited absorption or formulation properties can be key contributing factors.

We further applied our validated method to quantify JNJ in in vivo plasma and tissue samples. JNJ showed rapid absorption and exposure relative to IC_50_ values following a 10 mg kg^−1^ oral dose in balb/c mice. JNJ exhibited low plasma clearance (0.23 L h^−1^ kg^−1^) and extensive tissue distribution (V_d_ 1.2 L kg^−1^), consistent with the observed biodistribution data. Despite achieving total plasma concentrations exceeding the in vitro IC_50_ for MYC-driven MB cell lines, brain exposure was suboptimal. The low brain exposure may also indicate the influence of BBB transport mechanisms. We conducted PAMPA and Caco-2 studies, which primarily assess passive diffusion and do not fully measure active efflux processes at the BBB; therefore, further studies may be warranted to better characterize the role of transporter-mediated mechanisms in limiting CNS penetration. Although JNJ showed prolonged brain retention at later time points, the low K_p,uu,brain_ early after dosing indicates brain penetration was limited. Additionally, the increase in K_p,uu,brain_ at later time points may reflect delayed equilibration, tissue binding kinetics, or variability in measurement and warrants further investigation. The limited distribution is consistent with high protein binding, which also limits the free drug exposure at the site of action. We estimated that unbound plasma and brain concentrations/contents (Table 8) were substantially lower than the in vitro IC_50_ (more than 20-fold lower than IC_50_) across all time points, suggesting that high protein binding may limit pharmacologically active drug exposure, particularly in the brain.

In the context of the broader PRMT5 inhibitor landscape, these findings are consistent with class-level challenges. First-generation PRMT5 inhibitors have demonstrated robust target engagement, but mechanism-based hematologic toxicities, namely thrombocytopenia and anemia, often limit their therapeutic window [15,29,30]. In contrast, newer agents such as PRT811 and LLY-283 have been developed with improved brain penetration, with LLY-283 showing measurable CNS exposure and survival benefit in orthotopic glioblastoma models [11,31]. These comparisons suggest that JNJ’s limited ability to cross the BBB represents a key barrier to CNS exposure. While JNJ has a favorable metabolic and systemic PK profile, future research should focus on optimizing its bioavailability and brain exposure.

## 4. Materials and Methods

### 4.1. Chemicals and Reagents

JNJ (Figure 6) was purchased from MedChemExpress LLC (Monmouth Junction, NJ, USA), and oxibendazole, used as IS, was obtained from Sigma-Aldrich, St Louis, MO, USA, Mouse CD1 plasma was purchased from Innovative Research, Inc. (Novi, MI, USA). Mouse and human liver microsomes were purchased from Thermo Fisher Scientific (Frederick, MD, USA). β-Nicotinamide adenine dinucleotide phosphate reduced tetrasodium salt (NADPH) was purchased from MP Biomedicals (Irvine, CA, USA). Dialysis buffer for the protein binding assay was purchased from Thermo Fisher Scientific (Frederick, MD, USA). Freshly collected mouse plasma and brain tissue were collected from in-house animals. All other reagents were of analytical grade and purchased from typical vendors.

### 4.2. Animal

Male balb/c mice weighing 25–28 g purchased from The Jackson Laboratory (Farmington, CT, USA) were used for the PK and biodistribution study. All animal studies were conducted following the specific Institutional Animal Care and Use Committee (IACUC) protocol (Protocol no. 17-046-06-FC). All animals were housed in proper caging in an ambient atmosphere, maintaining a 12 h light/dark cycle with free access to food and water. Animals were given at least a time period of 3 days to acclimate to the conditions prior to doing the experimentation.

### 4.3. Stock, Calibration, and Quality Control Sample Preparation

A stock solution of JNJ was prepared at a concentration of 1 mg mL^−1^ in methanol. Working stock concentrations of 0.002, 0.005, 0.01, 0.02, 0.05, 0.2, 0.5, 2, and 5 µg mL^−1^ were prepared following a step-wise dilution approach and subsequently diluted during spiking into matrix to keep the final dynamic range of quantitation from 0.2 to 500 ng mL^−1^ in matrix. Four QC samples were prepared separately at final concentrations of LLOQ: 0.2 ng mL^−1^, LQC: 0.5 ng mL^−1^, MQC: 100 ng mL^−1^ and HQC: 375 ng mL^−1^. A stock solution of oxibendazole was diluted to 500 ng mL^−1^ using methanol to be used as an IS working stock. After preparation, all working stocks were stored at −20 °C for further use.

### 4.4. LC-MS/MS Instrumental Conditions

A Shimadzu Nexera UPLC (Ultra performance liquid chromatography) system integrated with an LCMS-8050 triple quadrupole mass spectrometer (Shimadzu Scientific Instruments, Columbia, MD, USA) was used for the quantitative analysis of the sample. For the chromatographic separation, a Synergy polar-RP80 Å (150 × 2 mm, 4 µm, Phenomenex, Torrance CA, USA) column was used. The mobile phase consisted of 0.1% formic acid in water (*v*/*v*) as the aqueous phase and methanol as the organic phase and operated at a flow rate of 0.25 mL min^−1^. The pumps were operated in gradient mode, and the elution program was set as follows: 0.1–3 min to reach from 35% to 65% organic; 3–5 min, from 65% to 90% organic; 5–8 min, hold at 90% organic; return to initial condition after 8.1 min and hold that for 2 min to condition for the subsequent injection.

For the MS/MS condition, ESI was operated in positive ion mode with MRM, and the working parameters were set as follows: nebulizing gas flow of 2 L min^−1^, drying gas flow of 10 L min^−1^, heating gas flow of 10 L min^−1^, interface temperature at 300 °C, desolvation line (DL) temperature of 250 °C and block heater temperature of 400 °C.

### 4.5. Method Validation

LC-MS/MS method validation was performed following the Food and Drug Administration (FDA) and ICH M10 guidelines for Bioanalytical Method Validation [26,32].

To evaluate the selectivity of the developed method, blank matrix was processed from six different sources alongside blank matrices spiked with LLOQ and HQC. If the response of the blank matrices was not more than 20% of the compound’s LLOQ or not more than 5% of the IS response in LLOQ, that represents the method’s suitability in differentiating the compound of interest from nonspecific matrix components.

Calibration curves were generated using nine calibration standard concentrations in the range 0.2–500 ng mL^−1^ by plotting the analyte-to-IS area ratio against the nominal concentration using a 1/x^2^ weighting and least-squares linear regression. The accuracy of the back-calculated concentrations was compared with that of the nominal concentrations, and if it was within ±20% for the LLOQ and ±15% for all the other levels, that signifies the method met the acceptance criteria.

Extraction recoveries of JNJ and IS were measured at the three concentration levels of LQC, MQC, and HQC. Calculation was done by measuring the peak area of the extracted samples upon the peak area of the post-extracted analyte spiked sample. Matrix effect was assessed by analyzing three replicates of LQC, MQC and HQCs spiked to the post-extracted matrix and then comparing the peak area of the post-extracted analyte spiked sample to that of the neat reference samples (prepared in mobile phase).

To evaluate the within-run and between-run accuracy and precision of the method, five QC samples were analyzed at three replicates at each concentration level in the same run and over five days. The accuracy level should be within ±20% for the LLOQ and ±15% for all other concentration levels. The %RSD, which represents the precision of the method, should be ≤15%, except at LLOQ, where it should be ≤20%.

Carryover was assessed by running a blank sample after analyzing the HQC to check any residual interference from the high-concentration analyte. The response in the blank sample should not exceed 20% of the compound’s response at the LLOQ and 5% response in the case of IS.

The stability of JNJ in mouse plasma was evaluated at three replicates of LQC and HQCs in different storage conditions. Studies were conducted with the study of bench-top stability for 4 h at room temperature, freeze–thaw stability (three freeze–thaw cycles), autosampler stability (4 °C for 48 h) and long-term stability (at −80 °C for 1 month). Samples were considered stable if the % drug remaining after storage conditions was within ±15% compared to the freshly prepared QC samples.

### 4.6. Sample Processing

Mouse plasma and tissue samples (liver, spleen, kidney, lung and brain) were prepared following the SPE procedure. Collected tissues were homogenized using Tissue Lyser II purchased from Qiagen Science, KY, after adding four times as much water as the tissue mass. For calibration standards and QC sample preparation, the 10 µL working stock and IS were spiked into 100 µL mouse plasma or tissue homogenates (liver, spleen, kidney, lung, and brain). After vortexing for 30 s on a Mixmate (Eppendorf North America, Enfield, CT, USA), samples were diluted with 600 µL of 5% formic acid and vortexed again at 1000 rpm for 2 min. Samples were centrifuged at 956× *g* for 10 min to get a clear sample that was not prone to blocking the cartridge during the SPE. Extraction was initiated by conditioning the cartridge (Oasis HLB 1 cc, Waters, Millford, MA, USA) with 1 mL of methanol, followed by a second conditioning with 1 mL of water. Then, the above-prepared samples were loaded into the cartridge and allowed to pass through the cartridge. Next, the cartridge was washed with 1 mL 15% methanol and dried for 1 min prior to the elution. For final elution, 500 µL 5% ammonium hydroxide in acetonitrile was eluted through the cartridges twice. Collected eluates were dried in a nitrogen dryer and reconstituted with 200 µL of mobile phase. Reconstituted samples were centrifuged at 956× *g* for 10 min, 150 µL supernatants were collected, and 5 µL was injected for LC-MS/MS.

### 4.7. Stability Studies

For the matrix stability study, JNJ was spiked into mouse plasma and brain homogenate at a final concentration of 5 µg mL^−1^ and incubated at 37 °C for 5 h on a shaking water bath in triplicate. At the specified sampling times, 50 µL aliquots were taken in the collection plate, followed by quenching with 300 µL acetonitrile containing 10 µL IS. After final collection, samples were vortexed at 1000 rpm for 2 min and centrifuged at 2200× *g* for 10 min, and the collected supernatant was analyzed using LC-MS/MS. Stability was estimated by determining the % remaining after 5 h of incubation using Equation (1).

(1) $$\%\;\mathrm{remaining}=\frac{{\mathrm{Concentration}}_{5h}}{{\mathrm{Concentration}}_{0h}}\times 100$$

### 4.8. In Vitro PK Studies

#### 4.8.1. Microsomal Stability

Mouse and human liver microsomes were used for the metabolic stability assay. In brief, the incubation mixture (500 µL) consisted of phosphate-buffered saline (PBS) (100 mM, pH 7.4), microsomal protein (0.5 mg mL^−1^), magnesium chloride (10 mM), and NADPH (2 mM). Following pre-incubation of the above reaction mixture for 10 min at 37 °C, the reaction was initiated by spiking JNJ at a final 1 µg mL^−1^ concentration. Alongside the test compound, testosterone at a final concentration of 1 µg mL^−1^ was incubated as a positive control. All experiments were performed in triplicate. At sampling times of 0, 5, 10, 20, 30, 45, and 60 min, 40 µL samples were collected and quenched with 300 µL ice-chilled acetonitrile supplemented with 10 µL IS. Simultaneously, samples from NADPH-free negative controls were also withdrawn and processed accordingly to confirm any non-enzymatic degradation. Finally, after 1 h of collection, samples were vortexed at 1000 rpm for 1 min and centrifuged at 2200× *g* for 15 min, and the collected supernatant was analyzed using LC-MS/MS.

The natural logarithm of the area ratio versus time was plotted, and the slope of the curve was calculated by a linear regression equation to generate the elimination rate constant, k (min^−1^). CL_int_ was calculated by the following equation:

(2) $${\mathrm{CL}}_{\mathrm{int}}=\frac{0.693}{t_{1/2}}\times \frac{\mathrm{Volume}\;\mathrm{of}\;\mathrm{incubation}\;\mathrm{mixture}\;(\mathrm{mL})}{\mathrm{microsomal}\;\mathrm{protein}\;(\mathrm{mg})}$$

CL_H,int_ of JNJ in the mouse and human liver was then estimated from CL_int_ using the scaling factor.

(3) $${\mathrm{CL}}_{H,\mathrm{int}}={\mathrm{CL}}_{\mathrm{int}}\times \frac{\mathrm{Microsomal}\;\mathrm{protein}\;(\mathrm{mg})}{\mathrm{Liver}\;(g)}\times \frac{\mathrm{Liver}\;\mathrm{weight}\;(g)}{\mathrm{Body}\;\mathrm{weight}\;(\mathrm{kg})}$$

Here, 45 and 32 mg of microsomal protein per g of liver weight were applied, and 60 and 25 g of liver weight per kg body weight were used for mouse and human tissue, respectively.

Then, CL_H,int_ was corrected for microsomal binding (CL_H,int_/f_u,inc_) and CL_H,predict_ was predicted using the well-stirred model [33] by the following equation:

(4) $${\mathrm{CL}}_{H,\mathrm{predict}}=\frac{Q\;\times \;f_{u,\mathrm{plasma}}\times \frac{{\mathrm{CL}}_{H,\mathrm{int}}}{f_{u,\mathrm{inc}}}\;}{Q\;+\;f_{u,\mathrm{plasma}}\times \frac{{\mathrm{CL}}_{H,\mathrm{int}}}{f_{u,\mathrm{inc}}}}$$

Here, Q is the hepatic blood flow (90 mL min^−1^ kg^−1^ in mouse), f_u,plasma_ is the fraction unbound in plasma, f_u,inc_ is the fraction unbound in microsomal incubation and CL_H,int_ is included from Equation (3); f_u,inc_ was calculated [34] by adding logP of JNJ (2.3) to the following equation:

(5) $$f_{u,\mathrm{inc}}=\frac{1}{1\;+\;10^{0.53\;\mathrm{logP}\;-\;1.42}}$$

#### 4.8.2. Plasma and Brain Protein Binding

A rapid equilibrium dialysis (RED) protein binding assay was performed to determine the protein binding of JNJ in plasma and brain matrix. The RED plate was obtained from Thermo Fisher Scientific (Frederick, MD, USA) for the experiment. An aliquot (200 µL) of JNJ spiked plasma/brain homogenate was poured into the donor chamber, and 400 µL dialysis buffer-PBS containing 100 mM sodium phosphate and 150 mM sodium chloride was added into the receiver compartment of each RED insert in triplicate. The plate was kept in incubation for 5 h on the orbital shaker at 37 °C. Following incubation, 50 µL plasma and buffer aliquots were collected separately, and an equal volume of opposite matrix was added. Collected samples were processed as previously described in Section 4.6 and analyzed using LC-MS/MS. Degree of protein binding (%) was calculated by the following equation:

(6) $$\mathrm{Protein}\;\mathrm{bound}\;(\%)=\frac{{\mathrm{Concentration}}_{\mathrm{donor}\;\mathrm{cell}}-{\mathrm{Concentration}}_{\mathrm{receiver}\;\mathrm{cell}}}{{\mathrm{Concentration}}_{\mathrm{donor}\;\mathrm{cell}}}\times 100$$

#### 4.8.3. Kinetic Solubility Study

An amount of 500 µL of PBS was taken into a 96-well plate in the required rows. The required volume of the JNJ stock solution was spiked to make the final concentrations of 160, 80, 60, 50, 40, 20, 10, and 5 µg mL^−1^. The plate was vortexed at 1000 rpm for 5 min and incubated at room temperature for 2 h. After incubation, aliquots of 100 µL of the samples were collected and diluted 2 times with acetonitrile. Diluted samples were centrifuged at 2200× *g* for 10 min. Then, 10 µL of supernatant was added to another collection plate, where previously 200 µL of the mobile phase supplemented with 10 µL IS was taken.

#### 4.8.4. Blood-to-Plasma Ratio (R_b/p_) Study

Freshly collected whole blood (400 µL) of balb/c mice was taken into an Eppendorf tube, and JNJ was spiked to obtain a final concentration at 5 µg mL^−1^ following proper mixing. Blood and plasma samples were collected at 0, 30, and 60 min and processed using the previously described extraction procedure. R_b/p_ was then estimated by dividing the JNJ concentration in blood by the concentration obtained in plasma samples separated from blood using Equation (5), where C_Blood_ is the drug concentration in blood and C_Plasma_ is the drug concentration in plasma.

(7) $$R_{b/p}=\frac{C_{\mathrm{Blood}}}{C_{\mathrm{Plasma}}}$$

#### 4.8.5. PAMPA Study

A Corning^®^ BioCoat™ Pre-coated PAMPA Plate System, obtained from Corning Life Sciences (Glendale, AZ, USA), was used for the noncell-based permeability assay. As per the manufacturer’s instructions, the PAMPA plate was warmed at room temperature for 30 min to make it ready for use. A JNJ stock solution was prepared in PBS at a concentration of 5 µg mL^−1^. Then, 300 µL of that prepared solution was poured into the donor side and 200 µL PBS was added to the acceptor side of each well in triplicate. After incubation for 5 h at room temperature, 50 µL samples were collected separately from the donor and acceptor chamber. Finally, samples were processed following the extraction procedure and analyzed using LC-MS/MS analysis. The apparent permeability coefficient is determined using the following formula:

(8) $$P_{\mathrm{app}}=\frac{-\ln[1\;-\;\frac{C_{a\left(t\right)}}{C_{\mathrm{eq}}}]}{A\;*\;\left(\frac{1}{V_{d}}\;+\;\frac{1}{V_{a}}\right)*t}$$

where P_app_ = apparent permeability in cm s^−1^, C_o_ = initial compound concentration in donor well (µg mL^−1^), C_d(t)_ = compound concentration in donor well at time t (µg mL^−1^), C_a(t)_ = compound concentration in acceptor well at time t (µg mL^−1^), V_d_ = donor well volume (0.3 mL), V_a_ = acceptor well volume (0.2 mL), A = filter area (0.3 cm^2^), and t = incubation time (18,000 s). C_eq_ is calculated by

(9) $$C_{\mathrm{eq}}=\frac{[C_{d(t)}*V_{d}+C_{a(t)}*V_{a}]\;}{\left(V_{d}+V_{a}\right)}$$

#### 4.8.6. Caco-2 Permeability Study

Human colon carcinoma cells (Caco-2) were obtained from ATCC (American Type Culture Collection) (Manassas, VA, USA). Caco-2 cells were cultured in Dulbecco’s Modified Eagle’s Medium (DMEM), supplemented with 10% fetal bovine serum, 1% nonessential amino acids, 100 U mL^−1^ penicillin, and 100 µg mL^−1^ streptomycin. The cells were split upon reaching the 80–90% confluence to make them ready to seed. Afterwards, the cells were seeded at a density of 80,000 cells/well in a 12-well tissue culture plate with a 0.4 µm pore size, purchased from CELLTREAT Scientific Products (Pepperell, MA, USA), and incubated at 37 °C in an incubator with 5% CO_2_ conditions. Following a culture of 21 days, the cell insert with the grown monolayer was washed with uptake buffer called Hank’s balanced salt solution (HBSS)–2-[4-2-(hydroxyethyl)-1-piperazinyl]ethane sulfonic acid (HEPES) twice before experimenting [35]. The integrity of the monolayer was evaluated by measuring the transepithelial electrical resistance (TEER) value using EVOM2™ Epithelial Voltohmmeter purchased from World Precision Instruments Inc. (Sarasota, FL, USA). Inserts that had a TEER value lower than 400 were rejected.

JNJ was spiked at a final concentration of 10 µM to the apical side in a volume of 500 µL HBSS-HEPES (10 mM) buffer along with the positive control (propranolol and atenolol) in respective wells. The basolateral chamber was filled with 1500 µL HBSS-HEPES buffer. After determined timepoints (0, 30, 60, 90, and 120 min), 100 µL aliquots were collected from the apical or basolateral sites, each time replacing with an equal volume of blank buffer. Collected samples were processed following the previously mentioned extraction procedure. The apparent permeability was calculated as follows to determine the apical to basal permeability of JNJ:

(10) $$P_{\mathrm{app}}=\frac{\mathrm{dQ}}{\mathrm{dt}}\times \frac{1}{A\;\times \;C_{0}\;}$$

where P_app_ = apparent permeability in cm s^−1^, dQ/dt = amount of product present in the basal direction (A-B) as a function of time (nmol s^−1^), A = area of transwell (1.12 cm^2^), and C_o_ = initial compound concentration in apical compartment (nmol mL^−1^).

#### 4.8.7. In Vivo PK Studies

For the oral PK study, male balb/C mice were grouped into two groups, with n = 4 for each group. Throughout the experiment, mice had free access to adequate food and water. The oral solution comprised 20% (*v*/*v*) polyethylene glycol 400, 10% (*v*/*v*) propylene glycol, 10% (*v*/*v*) ethanol, 2% (*v*/*v*) DMSO, 5% cremophore, and water as the rest of the volume. For the IV dose administration, 12 male balb/C mice were used in the study, divided into two groups. The IV solution used the same composition as the oral study. Doses of 10 and 2.5 mg kg^−1^ were used for oral and IV routes, respectively. Blood samplings were done at 5 min, 15 min, 30 min, 1 h, 2 h, 4 h, 8 h, and 24 h. After collection, blood samples were centrifuged immediately at 956× *g* for 10 min, and plasma was separated in an Eppendorf tube and stored at −80 °C until processed. In the oral PK study, tissues—liver, kidney, spleen, lung, and brain—were collected at 2, 8, and 24 h to assess the biodistribution pattern of JNJ in the oral route.

## 5. Conclusions

In summary, we developed and validated a suitable LC-MS/MS method for quantifying JNJ in plasma and tissue samples. While JNJ has entered phase I clinical studies, its complete initial preclinical PK, efficacy, and safety profiles are not reported in the public domain. This validated method can guide the accurate quantitative analysis of JNJ in future clinical studies. In this report, JNJ was identified as having druggable properties in terms of in vitro and in vivo studies. However, its limited brain penetrability and low oral bioavailability remain significant challenges for achieving adequate exposure. Future research is needed to optimize formulation strategies to enhance its brain penetrability and combat its low oral bioavailability.

## Figures and Tables

**Table 1:** List of MS/MS parameters: precursor ion, product ions, voltage potential (Q1), collision energy (CE), voltage potential (Q3), and retention time.

Analyte	MRM	Q1 (V)	CE (V)	Q3 (V)	Retention Time (min)
JNJ	484 > 135	−15	−35	−25	3.7
	484 > 236	−12	−44	−24	
Oxibendazole (IS)	250 > 218	−10	−18	−22	5.0
	250 > 175	−13	−27	−18	

**Table 2:** Accuracy (% bias) and precision (% RSD) of JNJ in mouse plasma.

		LLOQ (0.2 ng mL^−1^)	LQC (0.5 ng mL^−1^)	MQC (100 ng mL^−1^)	HQC (375 ng mL^−1^)
Accuracy (% bias)	Intra-day	2.5	7.6	−0.8	−8.4
	Inter-day	6.3	4.7	−8.3	1.8
Precision (% RSD)	Intra-day	4.3	7.6	1.5	1.8
	Inter-day	13.5	15.6	9.9	7.0

**Table 3:** Stability studies of JNJ in mouse plasma at different storage conditions.

QC Levels	% Stability Recoveries (Mean ± SD)			
	Bench-Top (RT, 4 h)	Autosampler (4 °C, 48 h)	Freeze–Thaw (−80 ± 5 °C After 3 Cycle)	Long-Term Stability (−80 ± 5 °C, 30 Days)
LQC	91 ± 9	110 ± 7	93 ± 4	97 ± 9
HQC	101 ± 15	110 ± 6	94 ± 8	95 ± 8

**Table 4:** The parameter estimates of JNJ in mouse (MLMs) and human liver microsomes (HLMs); (mean ± SD, n = 3).

Parameter Estimate	MLM	HLM
Half-life (t_1/2_, min)	101 ± 39	148 ± 10
CL_int_ (mL min^−1^ mg^−1^ protein)	0.02 ± 0.01	0.010 ± 0.001
CL_H,int_ (mL min^−1^ kg^−1^ body weight)	41 ± 19	7 ± 0.5
CL_H,predict_ (mL min^−1^ kg^−1^ body weight)	1.6 ± 0.7	-

**Table 5:** In vitro plasma and brain protein binding of JNJ (mean ± SD, n = 3).

Parameters	(Mean ± SD, n = 3)
Plasma Protein Binding (%)	97 ± 3
Brain Protein Binding (%)	94 ± 1

**Table 6:** Pharmacokinetic (PK) parameters of JNJ (mean ± SD, n = 4 for PO and 6 for IV at each time point). C_max_: maximum plasma concentration, T_max_: time of maximum plasma concentration, AUC: area under the curve, CL: plasma clearance, V_z_: volume of distribution, t_1/2_: terminal half-life.

PK Parameters	PO (Mean ± SD)	IV (Mean ± SD)
C_max_ (ng mL^−1^)	2781 ± 1033	4941 ± 1222
T_max_ (h)	0.42 ± 0.14	0.11 ± 0.08
AUC (ng·h mL^−1^)	6803 ± 1274	11,608 ± 3237
CL (L h^−1^ kg^−1^)	0.23 ± 0.04 *	0.23 ± 0.07
V_z_ (L kg^−1^)	0.88 ± 0.32 *	1.2 ± 0.7
t_1/2_ (h)	2.6 ± 0.7	3.3 ± 1.1
Bioavailability (%)	15 ± 5	N/A

* Oral CL and V_z_ have been normalized by bioavailability (%).

**Table 7:** JNJ tissue concentration after a single PO (10 mg kg^−1^) dose of JNJ in balb/c mice (mean ± SD, n = 4 at each time point).

	Content (ng g^−1^)				
Time (h)	Liver	Kidney	Spleen	Lung	Brain
2	6069 ± 1500	2952 ± 822	1900 ± 220	2570 ± 550	55 ± 27
8	1186 ± 167	474 ± 131	643 ± 58	599 ± 91	34 ± 1
24	52 ± 31	56 ± 27	95 ± 25	45 ± 25	23 ± 3

**Table 8:** Comparison of total and unbound plasma and brain concentrations/contents relative to in vitro IC_50_ and brain penetration parameters.

Time (h)	Brain Content (ng g^−1^)	Unbound Brain Content (ng g^−1^)	Plasma Concentration (ng mL^−1^)	Unbound Plasma Concentration (ng mL^−1^)	K_p,uu,brain_	In Vitro IC_50_ (ng mL^−1^)
2	55	3.1	1467	37	0.08	821
8	34	1.9	93	2.3	0.82	
24	23	1.3	1.6	0.04	33.3	

Unbound concentrations/contents were estimated using fraction unbound values in the plasma (f_u,plasma_ = 0.025) and brain (f_u,brain_ = 0.057). K_p,uu,brain_ represents the unbound brain-to-plasma partition coefficient.

## Data Availability

Data related to the findings of this study are available in the article.
